# Overexpression of steroidogenic acute regulatory protein in rat aortic endothelial cells attenuates palmitic acid-induced inflammation and reduction in nitric oxide bioavailability

**DOI:** 10.1186/1475-2840-11-144

**Published:** 2012-11-21

**Authors:** Dai Tian, Yanyan Qiu, Yongkun Zhan, Xiaobo Li, Xiuling Zhi, Xinhong Wang, Lianhua Yin, Yanxia Ning

**Affiliations:** 1 Department of Physiology & Pathophysiology, Shanghai Medical College, Fudan University, Shanghai, 200032, P.R. China

**Keywords:** Steroidogenic acute regulatory protein, Endothelial dysfunction, Palmitic acid, Inflammation, Lipid metabolism, Nitric oxide

## Abstract

**Background:**

Endothelial dysfunction is a well documented evidence for the onset of atherosclerosis and other cardiovascular diseases. Lipids disorder is among the main risk factors for endothelial dysfunction in these diseases. Steroidogenic acute regulatory protein (StAR), one of the cholesterol transporters, plays an important role in the maintenance of intracellular lipid homeostasis. However, the effect of StAR on endothelial dysfunction is not well understood. Palmitic acid (PA) has been shown to decrease eNOS activity and induce inflammation, both are the causes of endothelial dysfunction, in an endothelial cell culture model.

**Methods:**

StAR gene was introduced into primary rat aortic endothelial cells by adenovirus infection. Real-time PCR and Western blotting were performed to determine the relative genes and proteins expression level to elucidate the underlying mechanism. The free fatty acid and cholesterol quantification kits were used to detect total cellular free fatty acid and cholesterol. The levels of inflammatory factors and nitric oxide were determined by ELISA and classic Griess reagent methods respectively.

**Results:**

We successfully overexpressed StAR in primary rat aortic endothelial cells. Following StAR overexpression, mRNA levels of IL-1β, TNFα, IL6 and VCAM-1 and protein levels of IL-1β, , TNFα and IL-6 in culture supernatant were significantly decreased, which duing to blocke NFκB nuclear translocation and activation. Moreover, StAR overexpression attenuated the PA-induced reduction of nitric oxide bioavailability by protecting the bioactivity of pAkt/peNOS/NO pathway. Furthermore, the key genes involved in lipid metabolism were greatly reduced following StAR overexpression. In order to investigate the underlying mechanism, cerulenin and lovastatin, the inhibitor of fatty acid and cholesterol synthase, were added prior to PA treatment. The results showed that both cerulenin and lovastatin had a similar effect as StAR overexpression. On the other hand, the role of StAR was inhibited when siRNA was introduced to reduce StAR expression.

**Conclusions:**

Our results showed that StAR attenuated lipid synthesis and uptake as well as PA-induced inflammation and reduction in NO bioavailability in aortic endothelial cells. StAR can ameliorate endothelial dysfunction induced by PA via reducing the intracellular lipid levels.

## Background

The endothelium is considered to be the largest organ in the body. It is strategically located between the wall of blood vessels and the blood stream and is the major regulator of vascular homeostasis. Endothelium maintains the balance between 1) vasodilation and vasoconstriction, 2) inhibition and promotion of smooth muscle cell proliferation and migration, 3) prevention and stimulation of the adhesion and aggregation of platelets, and 4) thrombogenesis and fibrinolysis [1]. When the latter balance is disturbed, endothelial dysfunction occurs and causes damage to the arterial wall [2]. The observations made in human coronary arteries of atherosclerotic patients suggest that endothelial dysfunction is an early marker of atherosclerosis. The term “endothelial dysfunction” has been associated not only with atherosclerosis or hypertension, but also with other physiological and pathophysiological process, including aging, coronary syndrome, diabetes, impaired glucose tolerance, hyperglycemia, obesity, hypercholesterolemia, and inflammation etc [3]. A better understanding of various physiological and pathophysiological functions associated with endothelial cells will eventually lead to not only a better comprehension of these diseases but also improved preventive and therapeutic strategies.

Endothelial dysfunction is characterized by reduced dilator function, increased inflammatory cell and platelet adhesion, and increased coagulation activity [4]. Reduced bioavailability of nitric oxide (NO) is a major contributor of endothelial dysfunction [5]. Another potential trigger for endothelial dysfunction is inflammation. Inflammatory cytokines have been shown to impair endothelial function in animal models and isolated human veins [6].

Although there may be several drivers of endothelial dysfunction, the accumulation of intracellular lipids including triglycerides, cholesterol, and free fatty acid has emerged as a key pathophysiological factor [7-9]. Therefore, decreasing intracellular lipid levels in endothelial cells may be the key step for preventing or reversing endothelial dysfunction and related diseases, such as atherosclerosis, insulin resistance, and metabolic syndrome.

Steroidogenic acute regulatory protein (StAR) is one of the cholesterol transporters that initially found in steroidogesis tissues. It transfers cholesterol from the relatively cholesterol-rich outer mitochondrial membrane to the cholesterol-poor inner mitochondrial membrane for oxidation [10], which is the rate-limiting step in steroidogenesis. Recently, StAR had been demonstrated to be expressed in liver, endothelial cells other than the steroidogenic tissue with similar function [11]. Previous studies from our laboratory and others’ demonstrated that StAR is also expressed in human monocytes, human aorta [12], murine aorta [13], macrophages and endothelial cells [14]. Overexpression of StAR increased the mRNA and protein levels of ABCA1 and ABCG1 in microvascular endothelial cells [15], and decreased the cellular lipid levels and inflammation in macrophages [16]. In vivo investigations showed that StAR overexpression dramatically reduced cholesterol and triglyceride levels in serum, liver and aorta [17]. These evidences suggest that StAR plays a protective role in cardiovascular disease. However, the role of StAR in endothelial dysfunction remains unclear to date.

In the present study, the primary rat aortic endothelial cells (RAECs) were treated with palmitic acid (PA) as an *in vitro* model for endothelial dysfunction [18-20]. The effects of StAR overexpression on lipid metabolism, inflammation and NO bioavailability were investigated after infection with recombinant adenovirus encoding StAR in RAECs. We show here that StAR overexpression decreases intracellular lipid levels by reducing expression of genes involved in lipid metabolism. In addition, StAR overexpression inhibited PA-induced inflammation and attenuated impairment of NO bioavilability via regulating pAkt/peNOS/NO pathway. Overall our findings provided a strong support for StAR being as one of the key regulators for lipid metabolism and a protective molecule for endothelial dysfunctions in aortic endothelium.

## Methods

### Reagents

Culture media and reagents, fetal bovine serum, basic fibroblast growth factor and TRIZOL reagent were obtained from Invitrogen Life Technologies (Grand Island, NY). RevertAld™ First Strand cDNA Synthesis Kit and PageRuler Prestained Protein Ladder were purchased from Fermentas MBI (San Diego, CA). SYBR® Green real-time PCR Master Mix was from Bio-Rad (Hercules, CA). Primary antibodies against StAR, phosphor-eNOS (S1177) and eNOS were purchased from Abcam Ltd (Cambridge Science Park, Cambridge, UK). Antibodies against NF-κB (p65) and Histone (H3) were purchased from Proteintech Group, Inc (Chicago, IL). Phosphor-Akt (Ser473) was from Cell Signaling Technology, Inc (Boston, MA). Akt antibody was purchased from Bioworld technology, co, Ltd (Nanjing, Jiangsu, China). Primary antibody against b-actin and second antibodies against rabbit and mouse IgG were obtained from CWbiotech (Beijing, China). Cerulenin, an inhibitor of fatty acid synthase, was from Biovision (Milpitas, CA). Lovastatin, an inhibitor of HMG-CoA Reductase was purchased from Sigma-Aldrich Chemical Co (St.Louis, MO). siRNA for StAR gene and negative control were from Shanghai GenePharma., Ltd (Shanghai, China). The recombinant adenovirus encoding StAR (Ad-StAR) and the control adenovirus expressing the enhanced green fluorescence protein (Ad-EGFP) was a gift from Dr. Shunlin Ren (Dept. of Medicine, Veterans Affairs Medical Center and Virginia Commonwealth University, Richmond, VA). All other reagents were from Sigma-Aldrich Chemical Co unless stated otherwise.

### Cell culture

Rat aortic endothelial cells (RAECs) were isolated and cultured as described previously with minor modifications [21,22]. Briefly, segments of thoracic aorta were excised from male Wistar rats (150-180 g) and immediately placed in cold PBS containing 100 U/ml penicillin and 100 mg/ml streptomycin. The aorta was cut into 1 millimeter wide rings after the periadventitial fat was removed. Following transferred to a T-25 cm2 flasks (Nunc, Rochester, NY), the rings were cultured in Medium 199 containing 20% fetal bovine serum, 2.5 ng/ml basic fibroblast growth factors, 100U/ml penicillin and 100 mg/ml streptomycin. The aorta rings were placed at 37°C in a humidified atmosphere with 5% CO2 for 72-80 h without movement. All pieces of aorta rings were removed when cells migrated. Its microvascular cytological characteristics were demonstrated by CD31 and vWF staining as shown in the previous study [21]. In experiments involving PA treatment, M199 medium supplemented with 1% bovine serum albumin was used. All experiments were performed with RAECs up to passage 4. In the experiments with inhibitor, 5 μg/ml Cerulenin (in ethonal), or 5 μM lovastatin (in DMSO), or 3.3 μg/ml cerulenin plus 3.3 μM lovastatin was added in culture media 24 hours prior to PA treatment. The same volume of solvents was added at the same time as control. All experimental protocols were approved by the Animals Care and Use Committee of Shanghai Medical College, Fudan University which adopts the guideline for the care and use of laboratory animals published by the US National Institutes of Health (NIH Publication No. 85-23, revised 1996).

### Infection of cells with adenovirus encoding StAR

The RAECs were infected with recombinant adenovirus encoding StAR (Ad-CMV-StAR) as previously described [16,23]. Briefly, primary RAECs (4 × 10^5^ cells/well) were planted in 6-well plates (Costar, Corning, NY) in complete culture medium. Twenty-four hours after planting, RAECs were infected with recombinant adenovirus encoding Ad-CMV-StAR at a multiplicity of 10, 20, 50 or 100 pfu/cell. The recombinant adenovirus encoding enhanced green fluorescence protein (Ad-CMV-EGFP) was used as control. The virus was allowed to dwell for at least 2 h in minimal culture medium with shaking every 15 minutes. After 2 h of infection, unbound virus was removed and replaced with fresh medium. The cells were incubated for another 48 h before treatment. After determination the effect of infection, MOI = 10 were chose for the other experiment with sufficient overexperssion and minimum harm to the cells.

### Preparation of PA for vitro experiments

PA solution was prepared as described previously [24] with minor modifications. In brief, stock PA (0.1 M) was dissolved in 0.1 M NaOH at 70°C, and stored at -20°C. PA preparation was thawed and mixed with serum- and growth factor-free media in the presence of 1% BSA the day before use.

### Supression of StAR with siRNA

To down-regulate StAR mRNA expression, we transfected a siRNA specific for StAR and a non-coding control siRNA (GenePharma Ltd, Shanghai, China) using the Lipofectamine™ 2000 Transfection Reagent ( Invitrogen Life Technologies) according to the manufacturer’s protocol. Twenty-four hours later, cells were treated with PA and used for measuring gene expression (for inflammatory factor), and nitric oxide detection.

### Real-time quantitative PCR

To determine the effect of adenovirus infection, RAECs were seeded in 6-well plates and infected with Ad-CMV-StAR or Ad-CMV-EGFP for 48 h before harvest. To determine the effect of StAR overexperssion on the mRNA expression of inflammatory factors, RAECs were seeded in 6-well plates and treated with PA (200 μM) at different time (0 h, 1 h, 2 h, 4 h, 8 h, 12 h, 24 h) following with 48 h infection. RAECs were lysed and total RNA was prepared by TRIZOL reagent according to the manufacturer’s protocols. Complementary DNA (cDNA) was synthesized from 2 mg total RNA by RevertAld™ First Strand cDNA Synthesis Kit. Quantitative PCR was carried out in a CFX96 Touch™ Real-Time PCR Detection System (Bio-Rad, Hercules, CA) using SYBR® Green real-time PCR Master Mix. Reaction contained 20 ng of cDNA and 0.25 μM forward and reverse primers were performed as previously described [25]. The sequences of primers used in this study were listed in Table 1.

**Table 1:** The sequences of primers used in this experiment

**Gene**	**Forward primer (5′ → 3′)**	**Reverse primer (5′ → 3′)**
GAPDH	GCAAGTTCAACGGCACAG	GCCAGTAGACTCCACGACAT
Hu-StAR	CTGAGGCAACAGGCTGTGAT	AGCCGAGAACCGAGTAGAGAG
Rat-StAR	CACAGTCATCACCCATGAGC	AGCTCTGATGACACCGCTTT
ACC-1	CCCAACAGAATAAAGCTACTCTGG	TCCTTTTGTGCAACTAGGAACGT
FAS	CCTCTTCCCTGGCACTGGCTACCT	ACTCGGCGGGGATCGGGACTT
LDLR	AAGCCATTTTCAGTGCCAAC	AGGTGAACTTGGGTGAGTGG
HMGR	TGCTGCTTTGGCTGTATGTC	TGAGCGTGAACAAGAACCAG
SREBP-2	CATCTTCCCCTCTCCTTCCTAT	CCCAGCTTGACAATCATCTGC
IL-1β	CTGTGACTCGTGGGATGATG	GGGATTTTGTCGTTGCTTGT
TNFα	GCTACGGGCTTGTCACTC	CCACGCTCTTCTGTCTACTG
VCAM-1	ACAAAACGCTCGCTCAGATT	GTCCATGGTCAGAACGGACT
IL-6	CACAAGTCCGGAGAGGAGAC	ACAGTGCATCATCGCTGTTC

### Western blotting

Cells were grown in either 100 mm dishes or 60 mm dishes before lysis. To determine the transfection effects of adenovirus, cells were harvested after 48 h infection with different MOI adenovirus. To determine the effect of StAR overexperssion on pAkt/peNOS/NO pathway, cells were lysed after treating with 200 μM PA for 0, 30, 60 and 120 minutes following with 48 h infection. To determine the effect of StAR overexperssion on nuclear translocation of NFκB (p65), cytosolic and nuclear fractions were isolated and lysed after treating with 200 μM PA for 0, 15, 30, and 60 minutes following with 48 h infection. Cytosolic and nuclear fractions were isolated with Cytosolic and Nuclear Isolation Kit (Beyotime It. Co, Nanjing, China). Lysate preparation and analysis was done as previously described [16]. Fifty micrograms of total protein and thirty micrograms of cytosolic and nuclear was applied to SDS-PAGE gel electrophoresis and transferred to PVDF membrane (Invitrogen, Grand Island, NY) using a Bio-Rad transfer blotting system at 300 mA for 100 minutes. Proteins were probed with the following antibodies overnight at 4°C: StAR (1:2000), total Akt (1:1500), phospho-Akt Ser 473 (1:2000), total eNOS (1:1000), phospho-eNOS Ser 1177 (1:1000), and NFκB p65 (1:1500). To confirm equal loading of the gels, membranes were reprobed with β-actin (1:5000) for the whole lysates and Histon-H3 (1:1500) for nuclear fractions. Semi-quantitative analysis of protein expression was performed by densitometry using NIH ImageJ software (http://rsb.info.nih.gov/ij).

### Intracellular free fatty acid and total cholesterol determination

After 48 h infection, cells were harvested in PBS and lysated by sonication. Total intracellular cholesterol and free fatty acid were extracted by homogenization with 1/2 volume of chloroform/isopropanol/NP-40 (7/11/0.1, v/v) or chloroform-Triton X-100 (1% Triton X-100 in pure chloroform), respectively. The organic phase (lower phase) were collected and vacuumed dry to remove trace chloroform after spin the extracts at top speed in a microcentrifuge for 10 minutes. The extractions were redissolved and detected by Free Fatty Acid and Cholesterol Quantification Kit respectively (Applygen Technologies, Beijing, China) according to the manufacturer’s instructions.

### ELISA assay for cytokines

RAECs were treated by PA (200 μM) overnight (16 h) after 48 h infection and cell culture media were collected to determine the effect of StAR overexpression on secretion of cytokines. The cytokine levels (IL-1β, TNFα, and IL-6) were determined by ELISA assay (NeoBioScience, Shenzhen, China) following with the manufacturer’s instructions.

### Statistic analysis

Data are presented as the mean ± S.D. Statistical analysis was performed using *Student’s t* test or *ANOVA* as appropriate. *P* < 0.05 was considered statistically significant.

## Results

### StAR overexpression in RAECs by adenovirus infection

**Figure 1:**
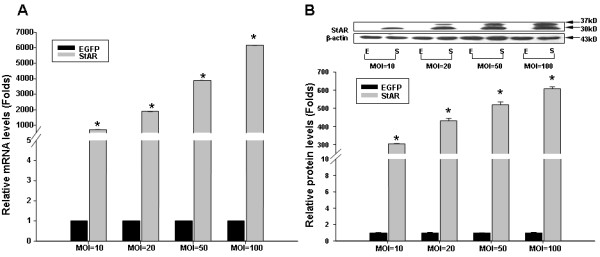
**StAR overexpression in RAECs by adenovirus infection.** Primary cultured RAECs were infected with the indicated recombinant adenoviruses as described in the “Methods” section. StAR mRNA and protein levels were determined by real-time Quantitative PCR (**A**) and Western blotting analysis (**B**). A: Real-time quantitative PCR analysis of StAR mRNA levels with adenovirus infection at different MOI (MOI = 10, 20, 50, 100) for 48 h. The data was normalized to internal GAPDH mRNA expression and presented as fold change. B: Western blotting analysis of StAR protein levels in RAECs after infection. β–actin serves as the loading control and a representative image is shown in upper panel. The data represent the means ± S.D. from three independent experiments and shown as fold change over the cells treated with Ad-CMV-EGFP. EGFP represents cells infected with recombinant adenovirus encoding CMV-EGFP and served as the negative control; StAR, cells infected with adenovirus encoding CMV-StAR; ^*^ represents *P < 0.05.*

StAR is a mitochondrial cholesterol transporter functioning in the process of steroid hormones synthesis [26]. Previous investigations have demonstrated that StAR is also expressed in liver cells [11] and endothelial cells [14]. However, our preliminary data showed that StAR expression level is quite low in RAECs. To investigate the effect of StAR on endothelial cells, we overexpressed StAR using recombinant adenoviruses carrying a CMV-driven gene encoding StAR (Ad-CMV-StAR) in RAECs. After infection, RAECs produced both a high StAR mRNA and protein level without inducing any cell toxicity. Real-time quantitative PCR analysis showed that StAR mRNA level increased by 708 to 6560 folds compared with the Ad-EGFP group using different virus multiplicity of infection (MOI) (Figure 1A). Western blotting analysis showed one major immunoreactive band with molecular weight of 30 kD and a second 37 kD band (Figure 1B) after infection, which is consistent with StAR premature and mature proteins as shown in the upper panel of Figure 1B. A summary of the data normalized to β-actin is shown in the lower panel (Figure 1B). Based on these results, we chose the MOI = 10 in the subsequent experiments with adequate overexpression effect and minimum toxicity.

### StAR overexpression inhibits expression of genes involved in lipid metabolism and reduces levels of intracellular free fatty acid and cholesterol

**Figure 2:**
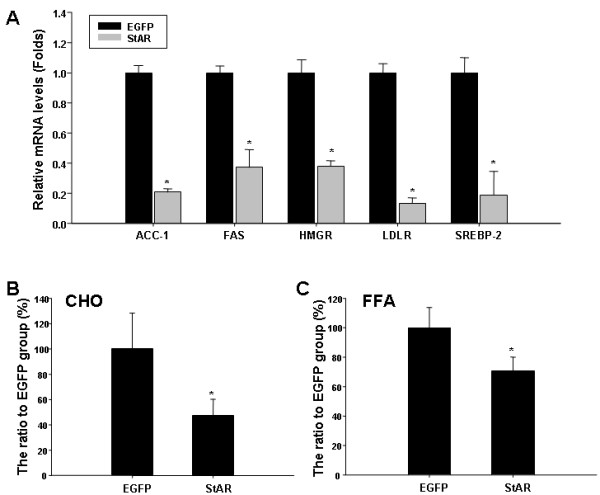
**StAR overexpression inhibited expression of genes involved in lipid metabolism and reduced levels of intracellular free fatty acid and cholesterol.** To determine the effect of StAR overexpression on lipids metabolism, the gene expression involved in lipid metabolism and intracellular total cholesterol and free fatty acid were detected after infected with adenovirus in RAECs. **A:** The mRNA levels of relative genes involved in lipid metabolism in RAECs infected with adenovirus. Data are presented as a ratio of gene-to-GAPDH expression (relative mRNA levels). RAECs were infected with adenovirus as indicated, and intracellular total cholesterol (**B**) and free fatty acid (**C**) were extracted and analyzed as described in the “Methods” section. Data represents the means ± S.D. from three independent experiments. ^*^ represents *P < 0.05.*

To determine whether StAR plays an important role in endothelial lipid metabolism, we first employed real-time quantitative PCR to analyze the expression of genes involved in lipid metabolism in RAECs received Ad-CMV-EGFP or Ad-CMV-StAR. Fourty-eight hours post transduction, the relative mRNA levels of ACC-1, FAS, HMGR, LDLR and SREBP-2 were decreased by 80%, 63%, 62%, 87% and 81% respectively in the Ad-CMV-StAR treated RAECs (Figure 2A), whereas the mRNA levels of SREBP-1, CYP27A1, PPARa and PPARg did not change significantly (data not shown). As demonstrated in previous studies, ACC-1, FAS, HMGR, SREBP-2 and LDLR are key enzymes involved in cholesterol and free fatty acids (FFAs) synthesis and endocytosis [27-30]. Thus, we next extracted intracellular lipids as indicated preciously [31]. Levels of cholesterol and FFAs were determined 48 h post infection. As shown in Figure 2B and C, intracellular total cholesterol and FFAs were decreased by 53% and 29% respectively after StAR overexpression compared with the Ad-CMV-EGFP group.

### StAR overexpression reduces inflammatory factors expression and secretion after palmitic acid treatment in RAECs

Numerous studies have shown that saturated FFAs could readily induce inflammatory response in endothelial cells via a NFκB-dependent pathway [32], and further cause endothelial dysfunction. Based on our previous results, we hypothesized that StAR plays a protective role in endothelial dysfunction induced by FFA via regulating lipid metabolism. Because palmitic acid (C16:0, PA) is a major component of dietary saturated fat representing up to 20% of the total FFA serum concentration [33], and has been shown to be present in a high percentage of atherosclerotic lesion [19], we therefore used palmitic acid combined with albumin to induce inflammation in RAECs.

**Figure 3:**
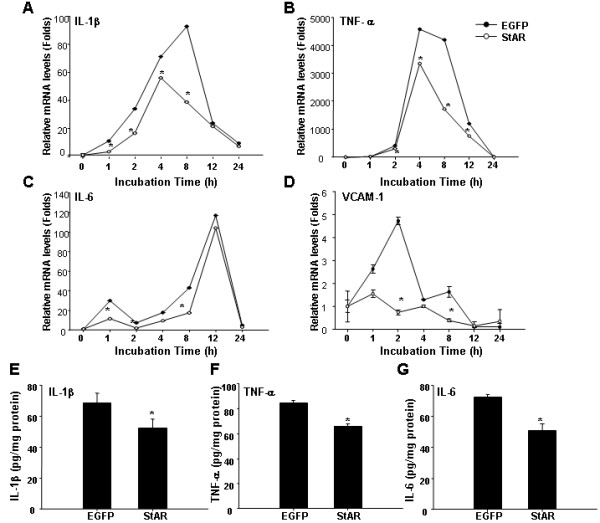
**StAR overexpression reduced inflammatory factors expression and secretion after palmitic acid treatment in RAECs.** RAECs were infected with Ad-CMV-EGFP or Ad-CMV-StAR at MOI = 10 for 48 h and treated with PA (200 μM) in time-course. (**A** through **D**) The mRNA levels of IL-1β, TNFα, IL-6, VCAM-1 were analyzed by real-time quantitative PCR. Data represented as a ratio of gene-to-GAPDH expression (relative mRNA levels) and fold change over the relative mRNA levels at 0 h. (**E** through **G**) The contents of IL-1β, TNFα, IL-6 in culture supernatant were detected by ELISA. Data represents the means ± S.D. from three independent experiments. ^*^ represents *P < 0.05.*

To determine the role of StAR on inflammatory responses induced by PA, adenovirus- transduced RAECs were treated with PA (200 μM) in a time-dependent manner. Cells were then harvested and total RNA was extracted for mRNA analysis. Real-time quantitative PCR analysis showed that the levels of IL-1β, TNFα, IL-6 and VCAM-1 increased significantly in a time-dependent manner after incubated with PA. At the same time, the mRNA levels of IL-1β, TNFα, IL-6 and VCAM-1 in cells with StAR overexpression were significantly lower than those in cells transduced with Ad-CMV-EGFP at any identical time (Figure 3, A-D).

To validate the changes in mRNA expression, culture supernatant were collected after treated with PA for 16 h and ELISA analysis were performed. As shown in Figure 3E, F and G, the contents of IL-1β, TNFα and IL-6 were decreased by 24%, 21%, and 30% after StAR overexpression.

### StAR overexpression inhibits PA-induced NFkB nuclear translocation in RAECs

**Figure 4:**
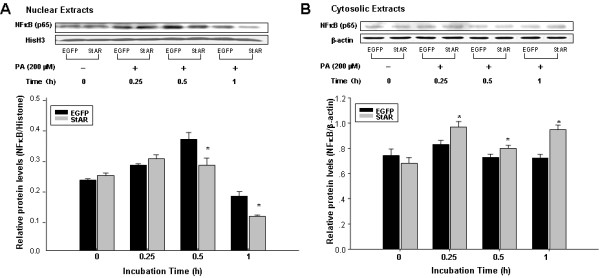
**StAR overexpression inhibited PA-induced NFkB nuclear translocation in RAECs. A**: Western blotting analysis of NFκB (p65) levels in the nuclear extracts. **B**: Western blotting analysis of NFκB (p65) levels in the cytosolic extracts. Histone H3 (HisH3) and β-actin were used as loading controls. Data represent the means ± S.D. for three independent experiments and shown as densitometry analysis. ^*^ represents *P < 0.05* vs. EGFP.

As shown in previous studies, the nuclear factor-κB (NFκB) transcription factor family has been considered the central mediator of the inflammatory process [34,35]. Translocation from cytosol to nuclear of p65/RelA, one of NFκB’s subunits, is the key step for NFκB activation [36]. To further investigate the underlying mechanism associated with StAR overexpression, we examined the nuclear translocation of NFκB p65 (RelA), which is the critical step for NFκB activation [37]. RAECs were treated with PA (200 μM) after adenovirus infection, and total cytosolic and nuclear proteins were extracted as described in the methods section for western blotting analysis. As shown in Figure 4A, the nuclear levels of NFkB p65 protein were significantly increased after PA treatment. In contrast, the nuclear levels of NFκB p65 were reduced by 23% and 37% after StAR overexpression at 0.5 and 1 h after PA treatment. Conversely, the cytosolic levels of NFκB p65 were higher in cells with Ad-CMV-StAR infection than those in cells received Ad-CMV-EGFP (Figure 4B). These results suggested that StAR overexpression blocked PA-induced NFκB p65 nuclear translocation in RAECs and further inhibited transcription of the inflammatory factors.

### StAR overexpression attenuates PA-induced reduction in nitric oxide bioavailability

**Figure 5:**
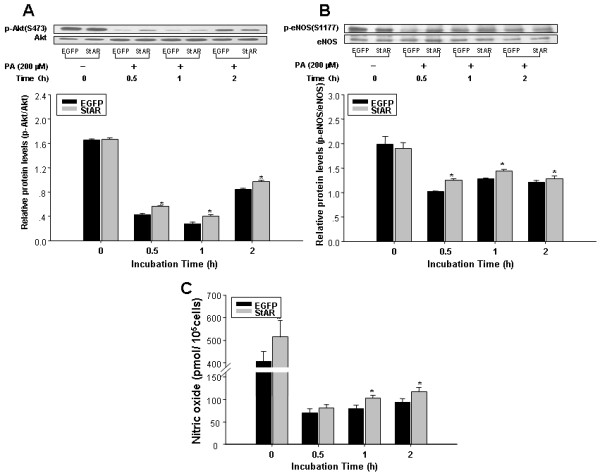
**StAR overexpression attenuated PA-induced reduction in NO bioavailability.** RAECs were treated with PA (200 μM) in a time-dependent manner after infected with adenovirus as indicated. The protein levels of phosphor-Akt Ser 473, total Akt, phosphor-eNOS Ser 1177 and total eNOS were detected by Western blot. The NO content in media supernatant was detected by Nitric Oxide Detection Kit. **A**: Western blotting analysis of phosphor-Akt Ser 473 and total Akt. **B**: Western blotting analysis of phosphor-eNOS Ser 1177 and total eNOS. β-actin was used as the loading control. Data represent the means ± S.D. from three independent experiments. **C**: NO contents in the culture supernatant. Data represents the mean ± SE from three independent experiments. ^*^ represents *P < 0.05.*

Excess PA has been shown to decrease eNOS activity in an endothelial cell culture model [20], and impair insulin-mediated NO production in human studies [38]. To investigate the effect of StAR overexpression on PA-induced downregulation of pAkt/peNOS/NO pathway, adenovirus-transduced RAECs were treated with PA (200 μM) in a time-dependent manner, and the protein levels of phosphor-Akt Ser 473 (pAkt), total Akt, phosphor-eNOS Ser 1177 (peNOS) and total eNOS were examined by Western blotting. PA treatment impaired the phosphorylation of Akt and eNOS in the absence of obvious changes in total Akt or eNOS protein levels. However, StAR overexpression attenuated the reduction of pAkt and peNOS levels compared to the control groups (Figure 5A and B). Consistent with the levels of eNOS phosphorylation/activation, NO bioavailability was also repressed with PA treatment, and this effect was partially blocked by StAR overexpression (Figure 5C).

### siRNA specific for StAR has the reverse effect as StAR overexpression on inflammatory factor mRNA expression and NO production

**Figure 6:**
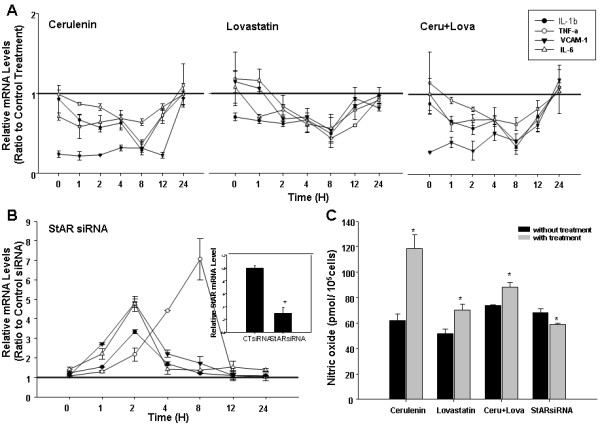
**Cerulenin and lovastatin has the similar effect while StAR siRNA has the reverse effect as StAR overexpression in inflammatory response and NO bioavailability.** Before treated with PA (200 μM), RAECs were treated with 5 μg/ml cerulenin (in ethonal), or 5 μM lovastatin (in DMSO), or 3.3 μg/ml cerulenin plus 3.3 μM lovastatin for 24 hours. In another experiment, RAECs were transfected with 100 nM siRNA specific for StAR for 24 hours. The mRNA levels of IL-1β, TNFα, VCAM-1 and IL-6 were detected by real-time quantitative PCR and NO content was detected by Nitric Oxide Detection Kit after PA treatment for 2 hours. **A**. The mRNA levels of IL-1β, TNFα, VCAM-1, IL-6 were analyzed by real-time quantitative PCR in cells pre-treated with indicial inhibitor. **B**. The mRNA levels of IL-1β, TNFα, VCAM-1, IL-6 were analyzed by real-time quantitative PCR in cells pre-treated with siRNA specific for StAR. Inset shows the confirmation of StAR mRNA reduction with siRNA transfection by real-time quantitative PCR. Data represented as a ratio of gene-to-GAPDH expression (relative mRNA levels) and ratio to the control treatment mRNA levels at any indicial time. Data represents the means ± S.D. from three independent experiments. **C**. NO contents in the culture supernatant. Data represents the mean ± SE from three independent experiments. ^*^ represents *P < 0.05.*

As shown previously, StAR overexpression could attenuate inflammatory response and NO production in RAECs treated by PA. In order to confirm the role of StAR, we performed the experiments with siRNA to knock-down StAR expression prior to PA treatment. The results indicated that StAR siRNA has the reverse effect as StAR overexpression on mRNA expression of IL-1β, TNFα, VCAM-1 and IL-6 and NO production (Figure 6B and C).

### Cerulenin, and lovastatin has the same effect as StAR overexpression on inflammatory factor mRNA expression and NO production

**Figure 7:**
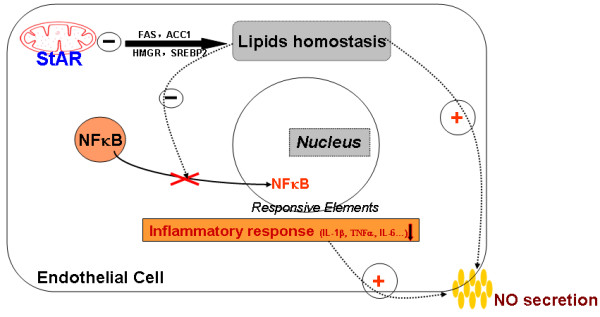
**The protective role of StAR in PA-induced endothelial dysfunction.** In rat aortic endothelial cells, the key genes involved in lipid metabolism are greatly reduced following StAR overexpression. As a result, the cellular levels of cholesterol and FFAs are also reduced after StAR overexpression, which maintains the homeostasis of lipids. Levels of mRNA expression and secretion of inflammatory factors were significantly decreased after PA treatment, which is due to the blockade of NFκB nuclear translocation and activation after StAR overexpression. Moreover, the reduction of NO bioavailability induced by PA are compensated by StAR overexpression.

As shown in previous studies, StAR plays an important role in lipid metabolism. To explore the underlying mechanism of StAR’s protective effect on endothelial dysfunction model, the inhibitor of fatty acid synthase and HMG-CoA reductase, cerulenin and lovastatin, were used before PA added. As shown in Figure 6A and C, the mRNA expression of IL-1β, TNFα, VCAM-1 and IL-6 were reduced while NO production was recovered with inhibitor treatment. This experiment demonstrated that the role of StAR on inflammatory response and NO production might due to its regulatory role in lipid metabolism. The schematic diagram was shown in Figure 7 to indicate the pathway of StAR in endothelial cells.

## Discussion

Vascular endothelial dysfunction is the initiation and hallmark of various cardiovascular diseases, such as atherosclerosis, hypertension, coronary vascular disease and diabetes. Several therapeutic interventions, including changes in lifestyle and pharmacologic treatments, are used to ameliorate endothelial dysfunction under various risk factors. It is of great interest to explore new therapeutic strategies to improve endothelial function for protection and prevention of these diseases which cause significant number of deaths each year.

In the present study, we provided insight into the effect of StAR on rat aortic endothelium lipid metabolism and dysfunction. As shown in Figure 1, StAR mRNA and protein levels were significantly increased by infection with recombinant adenovirus. Following StAR overexpression, key genes involved in fatty acid synthesis (FAS, ACC-1), cholesterol synthesis (HMGR) and uptake (LDLR, SREBP-2) were greatly repressed. And, intracellular total cholesterol and FFAs were significantly reduced. Furthermore, StAR overexpression inhibited mRNA expression and secretion of inflammatory factors by blocking NFκB nuclear translocation. Finally, StAR overexpression attenuated the reduction of NO bioavailability induced by PA treatment via regulating the p-Akt/p-eNOS/NO pathway.

An elevation of circulating FFAs is supposed to be related to the onset and progression of endothelial dysfunction [39,40], and associate with a number of cardiovascular risk factors, including hypertension, dyslipidemia, and abnormal fibrinolysis. It has been noted that FFAs may increase the production of multiple cytokines in mononuclear cells by generation of reactive oxygen species (ROS) and activation of the pro-inflammatory NFκB pathway in human endothelial cells [41,42]. In the present study, we used PA as one of the major mediators to induce endothelial dysfunction by activating inflammation and reducing NO biovailability.

When the endothelium in vessels is affected by risk factors, such as pro-inflammatory factors or hyperlipidemia, it will be activated and secrete inflammatory factors. Cell adhesion molecules including intercellular adhesion molecule-1 (ICAM-1), vascular adhesion molecule-1 (VCAM-1) and E-selectin are the biomarkers of endothelial activation [43]. As shown in our study, the mRNA expression and secretion of inflammatory factors were greatly increased after PA treatment in cultured rat aortic endothelial cells, while the mRNA expression of VCAM-1 was also upregulated significantly. However, the extent of increase is reduced when StAR is overexpressed by adenovirus infection. As shown by previous studies, the transcription factor NFκB regulates the expression of numerous genes including those related to pro-inflammatory responses, such as L-1β, IL-6, TNFα and VCAM-1, IL-1α [35,44]. We found that NFκB translocation from cytosol to nucleus was blocked by StAR overexpression. Thus, StAR could attenuate inflammatory responses in endothelium via its blocking of NFκB translocation.

There is strong clinical evidence that endothelial dysfunction contributes to the pathogenesis of cardiovascular disease and insulin resistance [45,46]. Endothelial cell dysfunction is defined by a decrease in the bioavailability of nitric oxide (NO), a critical regulator of vascular tone [47]. Endothelial nitric oxide synthase (eNOS) serves as a critical enzyme in producing NO. As shown in previous studies, the pAkt/peNOS/NO pathway was inhibited by PA treatment [19,20,46]. Our observations in this study demonstrated that StAR overexpression in endothelial cells attenuated the reduction of phosphorylation of Akt and eNOS, as well as NO production. Previous studies have shown an inverse correlation between StAR and NO in the steiodogenic tissues [48,49], in which NO was produced from activation of iNOS (also named NOS2). However, in our study, we observed a direct correlation between StAR and NO in vascular tissue, where NO is produced from eNOS (named NOS3). This suggests that StAR could take different roles in physiological process at different location. As shown in previous reports, NO production is regulated by a complicate network, including interactions among CaM, Cavelion-1, BH4 and ROS to regulate activation of eNOS (Ref). Each step in the network will affect the production of NO, and as a result, StAR overexpression ameliorates the reduction of NO production induced by PA treatment at a mild degree. Our results using siRNAs to inhibit StAR expression confirmed a positive role for StAR in endothelial dysfunction.

As already demonstrated in many investigations, lipids overload is a major risk factor for endothelial dysfunction. Our data obtained in the present study indicate that StAR inhibits lipid synthesis and uptake, PA-induced inflammation, and reduction in NO bioavailability in aortic endothelial cells. The inhibitor of FAS and HMGR could also ameliorate the inflammatory response induced by PA in RAECs. Taken together, the role of StAR overexpression in inhibiting inflammatory response and NO bioavailability in the cellular model of endothelial dysfunction might result from decreased lipid levels in RAECs.

## Conclusions

In conclusion, the present study demonstrated that StAR overexpression in RAECs decreased intracellular total cholesterol and FFAs levels. Furthermore, StAR overexpression inhibited PA-induced inflammatory response, and attenuated reduction in NO bioavailability. Taken together, these results indicate that StAR plays a protective role in PA-induced endothelial dysfunction via regulating intracellular lipid metabolism.

## Abbreviations

NO: Nitric oxide; StAR: Steroidogenic acute regulatory protein; FFA: Free fatty acid; RAECs: Rat aortic endothelial cells; PA: Palmitic acid.

## Competing interests

The authors declare that they have no competing interests.

## Authors’ contributions

DT initiated and performed the majority of the laboratory work, which was designed and supervised by YN; YQ, YZ, XL, XZ, and XW carried out additional experiments, including cell culture, transfection, and partial real-time quantitative PCR and Western blotting. LY and YN was critically involved in writing, revising, drafting the paper and has given final approval of the version of the paper to be published. All authors have read and approved the final manuscript.
